# Emerging Roles of the Intraflagellar Transport System in the Orchestration of Cellular Degradation Pathways

**DOI:** 10.3389/fcell.2019.00292

**Published:** 2019-11-19

**Authors:** Francesca Finetti, Nagaja Capitani, Cosima T. Baldari

**Affiliations:** Department of Life Sciences, University of Siena, Siena, Italy

**Keywords:** intraflagellar transport, T cell, degradation pathways, autophagy, lysosome

## Abstract

Ciliated cells exploit a specific transport system, the intraflagellar transport (IFT) system, to ensure the traffic of molecules from the cell body to the cilium. However, it is now clear that IFT activity is not restricted to cilia-related functions. This is strikingly exemplified by the observation that IFT proteins play important roles in cells lacking a primary cilium, such as lymphocytes. Indeed, in T cells the IFT system regulates the polarized transport of endosome-associated T cell antigen receptors and signaling mediators during assembly of the immune synapse, a specialized interface that forms on encounter with a cognate antigen presenting cell and on which T cell activation and effector function crucially depend. Cellular degradation pathways have recently emerged as new extraciliary functions of the IFT system. IFT proteins have been demonstrated to regulate autophagy in ciliated cells through their ability to recruit the autophagy machinery to the base of the cilium. We have now implicated the IFT component IFT20 in another central degradation process that also controls the latest steps in autophagy, namely lysosome function, by regulating the cation-independent mannose-6-phosphate receptor (CI-MPR)-dependent lysosomal targeting of acid hydrolases. This involves the ability of IFT20 to act as an adaptor coupling the CI-MPR to dynein for retrograde transport to the trans-Golgi network. In this short review we will discuss the emerging roles of IFT proteins in cellular degradation pathways.

## Introduction

Primary cilium assembly and function crucially relies on the intraflagellar transport (IFT) system. This multimolecular machinery, first described by Rosenbaum’s lab in the 1990s (Kozminski et al., 1995), allows for the crossing of proteins destined for the cilium through the physical barrier set by the transition zone at the base of the cilium and for their transport along the ciliary axoneme (Pedersen and Rosenbaum, 2008; Gonçalves and Pelletier, 2017). IFT depends on a wide array of IFT proteins that are organized into two complexes: the anterograde IFT-B complex, which interacts with kinesin to regulate the transport of molecules from the base to the tip of the cilium, and the retrograde IFT-A complex, which is required for the retrograde transport to the basal body through the interaction with dynein (Taschner and Lorentzen, 2016; Prevo et al., 2017). Both cytosolic and membrane-associated proteins are delivered to the cilium by the IFT system, which interfaces with a complex network of vesicular trafficking regulators (Pedersen et al., 2016).

Interestingly, evidence generated over the last 10 years indicates that the activity of the IFT system is not restricted to the primary cilium but contributes to key extraciliary functions, including cell cycle progression (Robert et al., 2007), mitotic spindle orientation (Jonassen et al., 2008; Delaval et al., 2011; Borovina and Ciruna, 2013), cleavage furrow formation (Qin et al., 2007; Wood et al., 2012; Taulet et al., 2017), microtubule and actin cytoskeleton dynamics (Bizet et al., 2015; Wang et al., 2016; Nishita et al., 2017), intracellular vesicular trafficking (Hsiao et al., 2012), and autophagy (Pampliega et al., 2013). The reader is referred to the thought-provoking review by Hua and Ferland (2018) for details regarding extraciliary roles for ciliary proteins. Consistent with these extraciliary functions, imaging studies have provided evidence that IFT proteins are localized not only at the base of the cilium and along the ciliary axoneme, but also in the cell cytoplasm. This is exemplified by IFT20, a large pool of which is associated with the Golgi complex and post-Golgi endosomal compartments (Follit et al., 2006, 2008; Finetti et al., 2009), and several other IFT proteins that have been detected in association with cytoplasmic vesicles in photoreceptor cells and in the postsynaptic terminals of non-ciliated secondary neurons (Sedmak and Wolfrum, 2010). Whether the extraciliary functions of the IFT system are carried out by IFT complexes with the same composition as those implicated in ciliogenesis remains to date a major open question.

**FIGURE 1:**
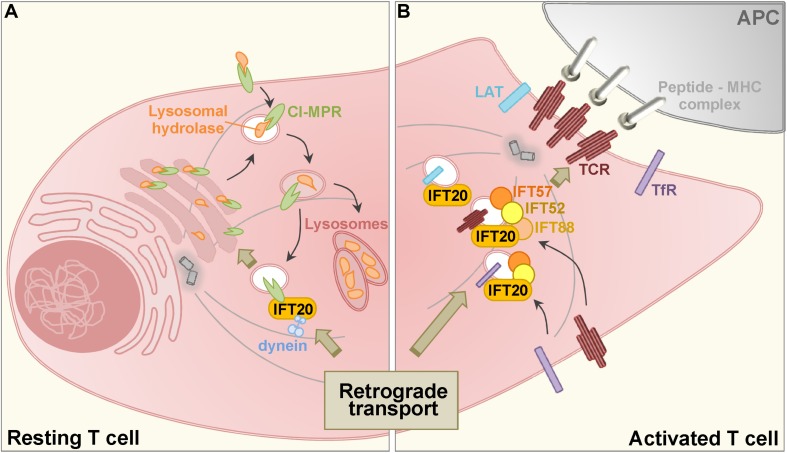
Schematic representation of the role of IFT20 in the retrograde transport in resting **(A)** and activated **(B)** T cells. **(A)** Acid hydrolases are added with mannose 6-phosphate moieties at the trans-Golgi network and bind to the cation independent mannose-6-phosphate receptor (CI-MPR) which is involved in their traffic from early to late endosomes (the decrease in pH is depicted by a color gradient, with darker vesicular lumen corresponding to the lowest pH). Here, the acidic pH leads to the dissociation of the complex: hydrolases are delivered to the lysosomes, while CI-MPRs recycle to the trans-Golgi through a retrograde transport pathway that requires IFT20 for coupling recycling CI-MPRs to dynein. **(B)** When a T cell encounter with an antigen presenting cell (APC) expressing specific peptide-MHC complexes, engaged T cell antigen receptors (TCRs) induce the assembly of the immune synapse. TCR as well as the transferrin receptor (TfR) and the membrane-associated adaptor LAT polarize to the T cell: APC interface from an intracellular endosome-associated pool through a retrograde transport pathway that requires IFT proteins.

The association of IFT proteins with endosomal compartments bears witness to their implication in intracellular vesicular trafficking, a role which had been initially proposed by Jékely and Arendt (2006) based on structural similarities with components of membrane coats revealed by bioinformatic analyses (Satir et al., 2008). We provided the first evidence of a cilium-independent, vesicular trafficking-related function of the IFT system in T lymphocytes which, similar to other hematopoietic cells, do not have a primary cilium (Finetti et al., 2009). We showed that IFT20 acts in concert with other IFT proteins to promote the assembly of the T cell immune synapse (Finetti et al., 2009), a specialized signaling platform that forms when a T cell encounters an antigen presenting cell that displays a specific peptide ligand bound to MHC and on which T cell activation crucially depends (Dustin and Choudhuri, 2016). This function involves the ability of IFT20 to interface with trafficking regulators and molecular motors to allow for the polarized delivery to the synaptic membrane of T cell antigen receptors and membrane-bound signaling mediators associated with recycling endosomes (Finetti et al., 2014; Onnis et al., 2016; Vivar et al., 2016; Figure 1B). We refer the reader to a recent review summarizing our current understanding of how T cells exploit IFT and other ciliary proteins for immune synapse formation (Cassioli and Baldari, 2019). Here, we will describe the emerging roles of the IFT system in intracellular degradation pathways, with a focus on recent findings by our lab that identify a new, trafficking-related function of IFT20 in the biogenesis of lysosomes.

## The IFT System in Cellular Degradation Pathways

Protein degradation through the ubiquitin-proteasome system (UPS) is essential for the assembly and resorption of the primary cilium (Kasahara et al., 2014). Among the targets of the UPS are centrosome-associated proteins, such as CSPP1, CP110, and CEP97, that negatively regulate ciliogenesis (Spektor et al., 2007; Tuz et al., 2014; Boukhalfa et al., 2019). Additionally, ubiquitin and ubiquitin ligases accumulate in flagella during resorption in the model organism *Chlamydomonas reinhardtii*, particularly in IFT mutants, indicating the UPS is active during cilia disassembly and that the ubiquitinated disassembly products are carried to the cell body through IFT (Huang et al., 2009). Moreover, the USP plays a critical role in signaling pathways controlled by the primary cilium, including the Sonic hedgehog, Notch and Wnt pathways, which involve the proteolysis-mediated activation of downstream transcription factors (Gerhardt et al., 2016). Consistent with this function, several ciliary proteins, including Bardet Biedl syndrome (BBS) 4, oral-facial-digital syndrome 1 (OFD1) and RPGRIP1L, co-localize with and interact with components of the proteasome at the base of the cilium. Interestingly, the UPS-dependent paracrine signaling involving IκB degradation and leading to activation of the transcription factor NF-κB was found to be controlled by BBS1, BBS4, and OFD1 (Liu et al., 2014), suggesting the possibility that ciliary proteins may have a role in cellular proteostasis beyond ciliary assembly/disassembly and signaling.

Interestingly, primary cilium assembly and function have been recently implicated in the other major proteostasis system of eukaryotic cells that is functionally interconnected with the UPS, namely autophagy. Here we will discuss the direct and indirect roles of the IFT system as a mediator of autophagy.

### Regulation of Autophagy by the IFT System

Autophagy is a selective process exploited by all eukaryotic cells to regulate cytoplasmic turnover of proteins and organelles. While the final degradation step is mediated by the lysosome, autophagy can be distinguished in macroautophagy, microautophagy, and chaperone-mediated autophagy based on the mechanism of cargo targeting to this organelle (Parzych and Klionsky, 2014). During chaperone-mediated autophagy a protein translocation system localized at the lysosomal membrane regulates the transfer of cytosolic proteins to the lysosomes, while during microautophagy autophagic substrates are internalized through lysosomal membrane invaginations. Macroautophagy (commonly simply referred to as autophagy) is the best characterized form of autophagy. This is a vesicle-based process that involves the formation of the phagophore, which encloses the autophagic substrates to generate a double membrane vesicle, the autophagosome. Subsequently, the autophagosome fuses with the lysosome to form the autophagolysosome, where the lysosomal hydrolases allow for degradation of its contents. Finally, aminoacids and other degradation products are exported to the cytoplasm by lysosomal permeases and transporters and used by the cell for biosynthetic processes (Yu et al., 2018).

Autophagy has been shown to control ciliogenesis, as demonstrated pharmacologically or genetically by depletion of the autophagy protein ATG7 (Wang et al., 2015; Pampliega and Cuervo, 2016). IFT20 and the ciliopathy-related protein OFD1 are among the ciliogenesis proteins which have been identified as targets of autophagy (Pampliega et al., 2013; Tang et al., 2013). Under nutrient-rich conditions IFT20 is selectively degraded by basal autophagy, thereby preventing ciliary growth. Conversely, under conditions of serum deprivation IFT20 is spared from degradation while the ciliogenesis inhibitor OFD1 undergoes autophagic degradation, allowing for ciliary growth (Pampliega et al., 2013; Tang et al., 2013). Interestingly, the ciliary protein RPGRIP1L, which modulates the activity of the ciliary proteasome at the base of the cilium (Gerhardt et al., 2015), also controls autophagy by regulating the activity of mTOR (Struchtrup et al., 2018), thereby acting as a rheostat in the activation of the mutually exclusive ciliogenesis and autophagy pathways.

While autophagy is orchestrated by the autophagy-related (ATG) proteins (Hurley and Young, 2017), several ciliogenesis proteins, including the IFT proteins, have been demonstrated to participate in this process. Namely, IFT20 and IFT88 control serum starvation-induced autophagy in a pathway involving Hedgehog signaling by regulating the transport of several components of the autophagic machinery to the ciliary base (Pampliega et al., 2013). Indeed, the intracellular distribution of autophagy regulators, the PI3 kinase VPS34, the ATG proteins ATG16L, ATG5, ATG7 and ATG14, and the ubiquitin-like proteins MAP1LC3/LC3 (microtubule-associated protein 1 light chain 3) and GABARAP, was found to be altered in IFT88^–/–^ mouse kidney epithelial cells, resulting in impaired autophagy (Pampliega et al., 2013). These results identify IFT proteins as regulators shared by the pathways that control autophagy and ciliogenesis and highlight a reciprocal interplay between these processes. Recruitment of the phagophore elongation complex component ATG16L to the basal body has also been observed in kidney tubular epithelial cells during fluid flow-induced autophagy (Orhon et al., 2016). The functional analysis of IFT20^–/–^ cells has led to the identification of IFT20 as responsible for this process. Indeed, based on co-immunoprecipitation and immunoimaging studies IFT20 was shown to interact with ATG16L and regulate its trafficking in association with carrier vesicles to the ciliary base (Pampliega et al., 2013), further underscoring the vesicular trafficking-related function of this IFT protein.

### IFT20 Controls Lysosome Biogenesis

Autophagy is exploited by T cells not only to regulate T cell homeostasis but also to support metabolic functions essential for T cell development, activation and differentiation to effector and memory cells (Puleston and Simon, 2014; Dowling and Macian, 2018; Jacquin and Apetoh, 2018). We thus hypothesized that, if the autophagic function described for the IFT system in ciliated cells is conserved in T cells, we could expect autophagy defects to contribute to the T cell activation and differentiation abnormalities displayed by IFT20-deficient T cells (Finetti et al., 2009; Vivar et al., 2016). We therefore measured the autophagic degradation activity in T cells depleted of IFT20, both under conditions of starvation and under nutrient and growth factor-rich conditions, using the standard immunoblot-based autophagy assay to measure the generation of LC3-II, the cleaved and lipidated form of LC3 that is associated with autophagosomal membranes (Klionsky et al., 2016). The results showed an autophagy defect, indicating that the autophagy-related function of IFT20 extends to the non-ciliated T cells (Finetti et al., 2019). Interestingly, an accumulation of LC3II could be detected in IFT20-deficient T cells under basal conditions (Finetti et al., 2019). Since LC3-II is degraded when autophagosomes fuse with lysosomes, this suggested the possibility of a defect in the late steps of the autophagy pathway in these cells. Indeed, imaging studies revealed that LC3-II accumulated in autolysosomes in IFT20-depleted T cells, concomitant with the lysosomal accumulation of lipid droplets, which similar to LC3-II are degraded by lysosomal hydrolases (Singh et al., 2009; Platt et al., 2012; Yu et al., 2018). These results pointed to a lysosomal defect, one consequence of which would be impaired autophagy.

IFT20 deficiency in T cells resulted in a peculiar lysosome phenotype, with a decrease in their number which was paralleled by an increase in their size. This is the typical phenotype observed with dysfunctional lysosomes that become engorged by undigested material. Under these conditions the cell attempts to compensate by promoting the biogenesis of new lysosomes through the activation of the CLEAR (coordinated lysosomal expression and regulation) gene network. This includes all the genes that encode the structural and effector components of the lysosomes and is controlled by the master transcription factor TFEB (Garbi et al., 2010; Palmieri et al., 2011; Settembre et al., 2011). In support of a lysosomal dysfunction, the expression of genes belonging to the CLEAR network was upregulated in IFT20-deficient T cells, including the integral lysosomal membrane proteins LAMP-1 and -2, the proteases cathepsin B and D, the glycosaminoglycan-degrading enzyme hexosaminidase B, the lysosomal acid lipase and TFEB itself (Finetti et al., 2019).

Lysosome function depends on two main factors: acidification, which is mediated by the vacuolar ATPase and is essential for the activation of the lysosomal hydrolases, and transport of these hydrolases to the lysosomal lumen. Acidification was normal in IFT20-deficient T cells, as assessed using a pH sensitive probe. However, lysosomal protease activity was impaired, as measured using acid protease fluorescent probes, suggesting that the transport of the lysosomal hydrolases might be affected by IFT20 deficiency. In support of this notion, we found that lysosomes purified from IFT20-deficient T cells had a significant reduction in their acid hydrolase content (Finetti et al., 2019).

The intracellular trafficking of lysosomal enzymes relies on the activity of the cation-independent mannose 6-phosphate receptor (CI-MPR), a type-I 300-kDa transmembrane protein, which diverts these enzymes from the default secretory pathway by binding them, once they have been N-glycosylated and then added with mannose-6-phosphate at the trans-Golgi network (TGN), where they are sorted into clathrin-coated vesicles. Vesicles transit through early endosomes to late endosomes, where the low pH allows for the dissociation of CI-MPRs from the hydrolases, which are released into lysosomes by fluid-phase transport, while CI-MPRs undergo retrograde traffic to the TGN for new cycles of transport (Braulke and Bonifacino, 2009; Lu and Hong, 2014). Using a specific antibody to tag CI-MPRs undergoing retrograde transport, we observed that these fail to efficiently recycle to the TGN in IFT20-deficient T cells and remain associated with endosomes dispersed away from the centrosome, implicating IFT20 in the retrograde transport of the CI-MPR (Finetti et al., 2019).

The CI-MPR retrieval pathway requires the retromer complex, which sequestrates the receptor into tubular endosomes, several Rab GTPases and their effectors, and the STX16/Vti1A/VAMP-3 SNARE complex, as well as the interaction with the dynein/dynactin minus-end directed microtubule motor complex (Hong et al., 2009; Wassmer et al., 2009; Cai et al., 2010; Pfeffer, 2011; Lu and Hong, 2014). Since one of the basic functions of the IFT system in ciliated cells is to couple cargo to molecular motors for transport along the axonemal microtubules we tested the association of IFT20 with dynein. We found that IFT20 co-precipitated and co-localized with dynein and the CI-MPR and that the ability of the CI-MPR to interact with dynein was impaired in IFT20-deficient T cells (Finetti et al., 2019).

Together, these findings indicate that IFT20 regulates CI-MPR trafficking by coupling this receptor to dynein to allow for its retrograde transport to the TGN (Figure 1A). In its absence CI-MPRs accumulate in a dispersed traffic-incompetent endosomal compartment, thereby limiting the availability of CI-MPRs at the TGN. This leads to a defect in the transport of acid hydrolases to lysosomes and impaired lysosome function. Additionally, these data highlight IFT20 as an adaptor implicated not only in endosome recycling to the plasma membrane, but also as a new component of the machinery responsible for retrograde transport to the TGN (Finetti et al., 2019). Consistent with this function, IFT20 participates in the endosome-mediated transport of the molecular adaptor LAT to the immune synapse (Vivar et al., 2016), which has recently been shown to depend on its Rab6-depedent retrograde transport to the Golgi apparatus (Carpier et al., 2018).

Of note, we have extended this study to B cells, which similar to T cells have no primary cilium, as well as to fibroblasts, which have a cilium. The lysosome-related defects observed on T cells were found to be shared by these cells when depleted of IFT20 (Finetti et al., 2019), underscoring the implication of IFT20 in a fundamental extraciliary function. Interestingly, in sperms from a male germ cell-specific IFT20^–/–^ mouse redundant cytoplasm was observed attached to the flagella, concomitant with a reduction in mature lysosomes, implicating IFT20 in the degradation of cytoplasmic components of the residual bodies in mouse testis (Zhang et al., 2016). Together, these data highlight a new cellular function of IFT20 in intracellular membrane trafficking related to the cellular degradation pathways that is conserved in ciliated and non-ciliated cells.

## Conclusion and Future Directions

The emerging scenario is that, by orchestrating intracellular vesicular trafficking, IFT proteins control crucial extraciliary processes spanning from endosome recycling to lysosome biogenesis, and function. These findings have important implications for the development of an effective immune response. We have previously reported that T cell activation and differentiation are impaired in T cells lacking IFT20 both *in vitro* and *in vivo* in a T cell-specific conditional knockout mouse and showed that this results from its ability to promote the polarized recycling to the immune synapse of endosome-associated TCR and LAT (Finetti et al., 2009; Vivar et al., 2016). The recent identification of IFT20 as a central regulator of lysosome function (Finetti et al., 2019) opens new future perspectives on its ability to control the immune response at a more global level. One central question is whether the lysosomal defect in IFT20-deficient T cells might impinge not only on the activation but also on the activity of cytotoxic T cells, which are responsible for the elimination of infected or cancer cells. Indeed, lytic granules are specialized lysosomes that exploit the CI-MPR pathway for the transport of the granzymes and possibly of perforin (Clark and Griffiths, 2003; Lopez et al., 2012), which mediate killing of their specific cell targets. Future studies will be required to evaluate the implication of IFT20 in the biogenesis of these organelles in cytotoxic T cells as well as in NK cells, their innate cytotoxic counterparts. Another level at which IFT20 may indirectly impact on the generation of T cell specific immunity through its lysosome-related function is by modulating the ability of antigen presenting cells to activate T cells. Indeed, antigen processing and presentation through the MHCII pathway, on which the generation of T cell-mediated immunity is critically dependent, also requires functional lysosomes, suggesting that IFT20 might participate in this process. Finally, lysosomal integrity has been demonstrated to be required for efficient extraction of surface-tethered antigens at the synapse formed by B cells with follicular macrophages, as this process relies on the local secretion of lysosomes that release lytic enzymes and acidify the synaptic cleft, allowing for antigen extraction (Yuseff et al., 2013; Sáez et al., 2019). Hence, by modulating lysosome function IFT20 may also participate in B cell activation. These considerations suggest new exciting directions to be tested experimentally to elucidate the role of IFT20 and potentially other IFT proteins that physically and/or functionally interact with IFT20 in the lysosomal control of the immune response. Additionally, these observations highlight the potential involvement of IFT proteins in T and B cell-related immunodeficiency disorders of unknown etiology and also underscore the importance to search for the presence of immune dysfunctions in ciliopathies that have been linked to mutations in components of the IFT system or of other ciliary proteins implicated in the IFT-dependent pathways of lymphocyte activation.

Another important implication of the lysosome-related function of IFT20 and, at a more general level, of the extraciliary functions identified to date for ciliary proteins, is that the complex multiple abnormalities observed in the wide range of known ciliopathies and attributed to defects in cilia assembly and/or function may need to be reconsidered at the light of these functions. In this respect, the non-ciliated immune cells represent a perfect system to study cilium-independent cellular functions.

## Author Contributions

All authors listed have made a substantial, direct and intellectual contribution to the work, and approved it for publication. FF, NC, and CB wrote the manuscript. FF prepared the figure.

## Conflict of Interest

The authors declare that the research was conducted in the absence of any commercial or financial relationships that could be construed as a potential conflict of interest.
